# How the Great Plains Dust Bowl drought spread heat extremes around the Northern Hemisphere

**DOI:** 10.1038/s41598-022-22262-5

**Published:** 2022-10-17

**Authors:** Gerald A. Meehl, Haiyan Teng, Nan Rosenbloom, Aixue Hu, Claudia Tebaldi, Guy Walton

**Affiliations:** 1 National Center for Atmospheric Research, Boulder, CO USA; 2 PNNL, Richland, WA USA; 3 PNNL, Washington, DC USA; 4 Weather Channel, Atlanta, GA USA

**Keywords:** Climate sciences, Atmospheric science

## Abstract

Extraordinary heat extremes occurred in the 1930s in areas of the Northern Hemisphere far from the record setting heat over the US associated with the Great Plains Dust Bowl drought. A climate model sensitivity experiment is used to identify a new mechanism involving a warm season circumglobal atmospheric teleconnection pattern that spread heat extremes over far-flung areas of the Northern Hemisphere arising from the intense heating over the desiccated Great Plains themselves. It has only been in the twenty-first century that human populations in these regions of the Northern Hemisphere have experienced heat extremes comparable to the 1930s. This demonstrates that humans influenced Northern Hemisphere temperature and heat extremes through disastrous and unprecedented regional land use practices over the Great Plains, and points to the possibility that future intense regional droughts could affect heat extremes on hemispheric scales.

The Dust Bowl Drought of the 1930s (1932–39) occurred over the Great Plains of North America and was one of the worst natural disasters of the twentieth century^1^. It was associated with anomalously warm temperatures over the U.S. that have only recently been equaled or exceeded (Fig. 1a). It is thought that the drought was initiated and maintained by a combination of decadal timescale internally-generated negative sea surface temperature (SST) anomalies in the tropical Pacific associated with the negative phase of the Interdecadal Pacific Oscillation (IPO), and positive SST anomalies north of the equator in the Atlantic with the positive phase of the Atlantic Multidecadal Oscillation (AMO)^2,3^. The resulting convective heating anomalies in those two basins then forced atmospheric circulation anomalies that produced a naturally-occurring drought over the Great Plains^2–4^ along with anomalous heat^5^ and associated heat extremes there^6,7^.

**Figure 1:**
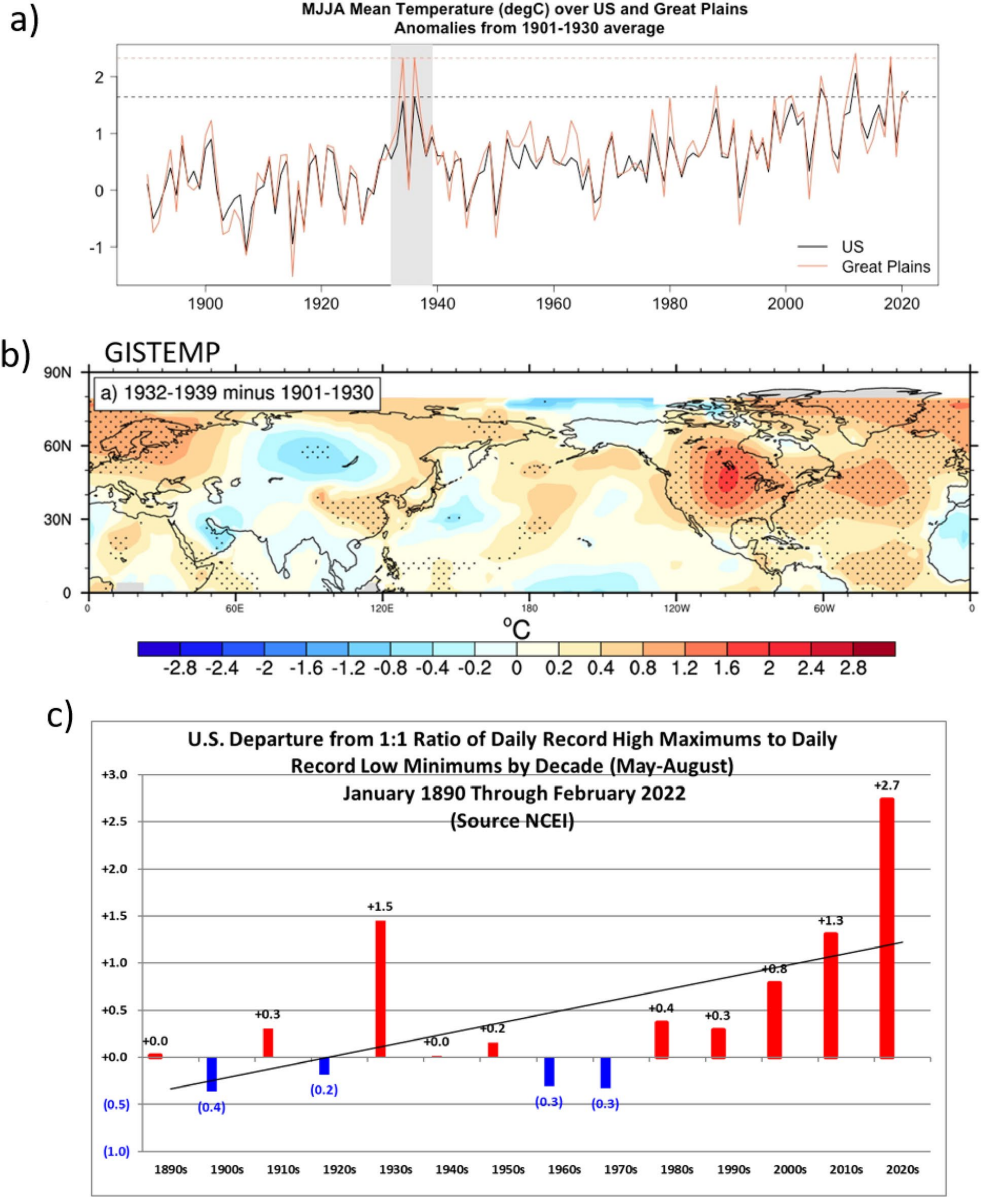
The warm 1930s and heat extremes. (**a**) Season average MJJA mean surface air temperature anomalies (°C) relative to 1901–1930 over the continental U.S. (black line) and the Great Plains (red line) using the BEST observations. Gray shading denotes the Dust Bowl years between 1932 and 1939, and horizontal lines indicate the maximum MJJA value during that period (occurring in 1936) for both U.S. (dashed black line) and Great Plains (dashed red line); (**b**) season average MJJA mean surface air temperature anomalies (°C), 1932–39 minus 1901–1930, from GISTEMP; stippling denotes differences significant at the 95% level; (**c**) decadal averages over the continental U.S. of the ratio of daily record high maximum temperatures to record daily record low minimum temperatures from NCEI, depicted as deviations from the nominal value of 1.0 (the value of the ratio with no change to either record highs or record lows). To obtain the total ratios, add the nominal value of 1.0 to the values plotted so that the total records ratio for the 1930s would be 2.5, with the deviation from 1.0 to 1.5.

Similar conditions have been responsible for other Great Plains droughts (e.g. in the 1950s^8^) though those droughts were not nearly as severe as the Dust Bowl drought. The Dust Bowl was likely made worse by unprecedented and disastrous land use practices where most of the grassland ecosystem in the Great Plains was plowed up to plant dryland wheat to supply the enhanced demand during WWI that produced record high prices^1^. When a naturally occurring drought occurred during the 1930s with the coincidental onset of the Great Depression, wheat prices plummeted and farmers desperately plowed up millions more acres to survive^1^. But there was not enough precipitation to sustain crops that could have served as ground cover. The bare soil dried up and, as evapotranspiration plummeted, surface temperatures soared and the desiccated soil yielded tremendous dust storms. The combination of dryness and dust is thought to have intensified the drought and associated heat over the Great Plains to a level not seen again until the modern era (Fig. 1a)^6,8,9^. However, the anomalous heat associated with the Dust Bowl was not confined just to the Great Plains. Unusually high surface temperatures also occurred over most of North America and in other regions of the Northern Hemisphere, particularly over Northern Europe, and parts of eastern and northeastern Asia (Fig. 1b).

We apply a measure of heat extremes represented by the ratio of daily record high maximum temperatures to record low minimum temperatures^10^. To calculate this index, we sum the number of daily record high maximum temperatures (at each station in the observations) for each year averaged by country, and also sum the number of daily record low minimum temperatures in the same way. We then compute the ratio of the sum of the record highs divided by the sum of record lows, and form the ratio averaged by country and by year. We then perform decadal averages of the ratio. If the background climate is relatively stable, there would be an equal chance of setting a daily record high maximum or daily record low minimum at a given location, and the ratio would be around one. However, if the mean climate is anomalously warm, the odds are shifted towards a greater chance of record highs compared to record lows, and the ratio of record highs to record lows goes above one^10^. Over the U.S. there is a correlation of + 0.62 between the records ratio and average temperatures, such that higher average temperatures produce higher records ratios indicative of more extreme heat^11^. If the records ratios from weather stations over the U.S. for the warm season (May–June-July–August, MJJA) are compiled by decade, the 1930s stand out (Fig. 1c) with a records ratio of 1.5 above the nominal value of 1 for a total ratio of 2.5. This magnitude of records ratio for the warm season has not been seen in subsequent decades again over the U.S. As average warming has increased mainly from ongoing increases of increasing greenhouse gases (GHGs), the records ratio after the 1930s has risen, with the total value of 2.3 for the 2010s coming closest to the 1930s. There are indications of even larger values so far in the 2020s (Fig. 1c).

The large records ratios over the U.S. in the 1930s, when there was a much smaller forcing from increasing GHGs, indicates that something else was contributing to much of that extreme heat. A viable candidate is the Dust Bowl Drought itself^6^. This is plausible because the low values of evapotranspiration associated with the bare and desiccated soil during the drought over the Great Plains would produce warmer mean temperatures and greater heat extremes as noted above in other studies. However, heat extremes also stood out in the 1930s in other areas of the Northern Hemisphere corresponding to the previously mentioned increases of mean temperature (Fig. 1b). Records ratios were well above one in other regions of North America such as Canada (total ratio for MJJA of 2.0, Fig. 3a), and in areas far from the Great Plains such as Sweden (total ratio of 2.9, Fig. 3b), Germany (total ratio of 1.6, Fig. 3c) and Russia (with a total ratio of 1.5, Fig. 3d). The significant surface warming in areas of eastern China would suggest large records ratios there as well during the 1930s, but the available data for daily temperature records in China do not go back as far as the 1930s. In any case, since the areas in Fig. 2 lie in areas of the Northern Hemisphere well outside the Dust Bowl region of the Great Plains, the implication is that other mechanisms produced the anomalous mean warmth and consequent heat extremes in those regions in the 1930s.

**Figure 2:**
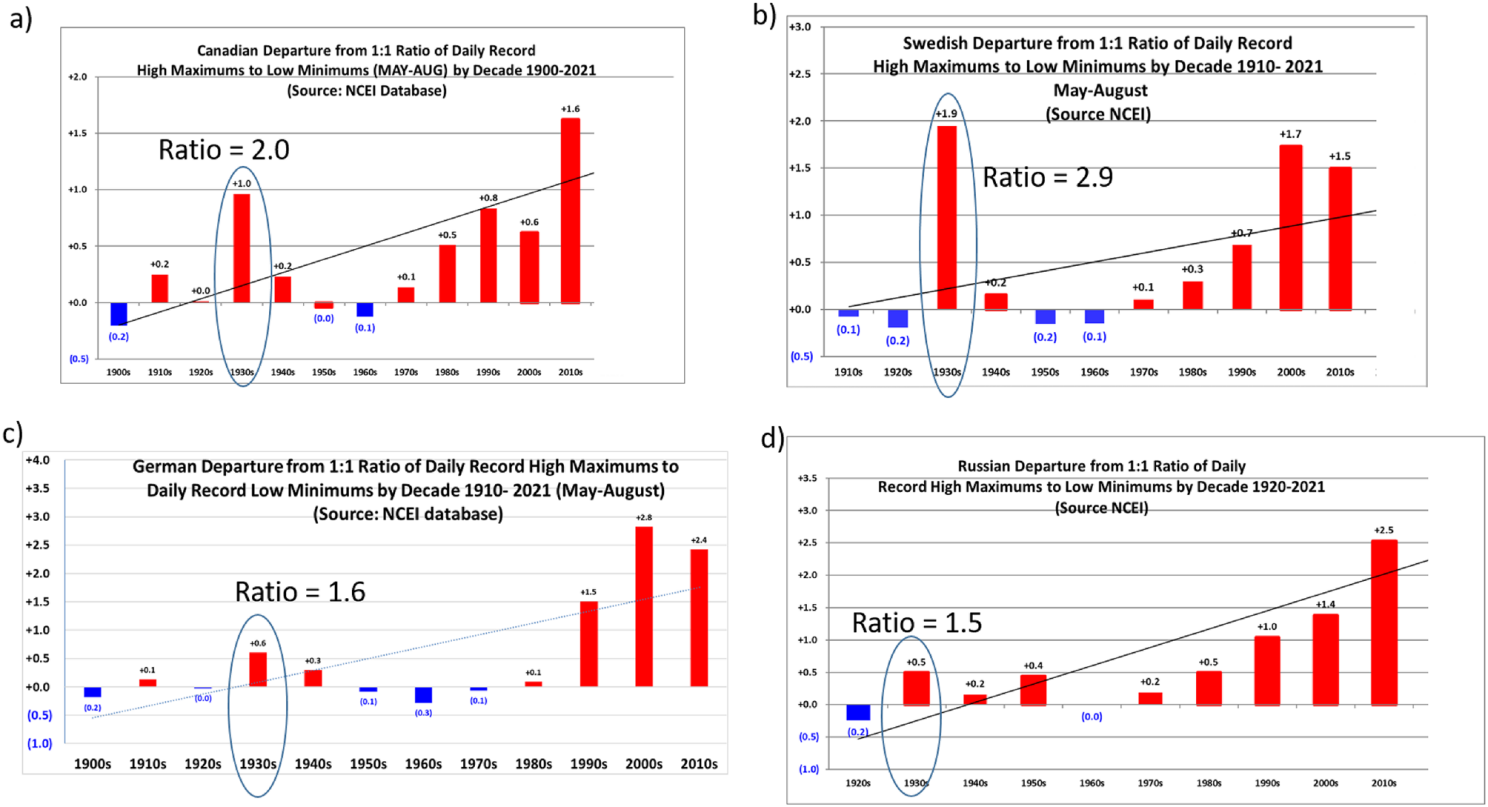
Extreme heat in the 1930s far from the Great Plains. Same as Fig. 1c, except for country values by decade for (**a**) Canada, (**b**) Sweden, (**c**) Germany, and (**d**) Russia. The total records ratios for the 1930s stand out for most of the twentieth century and are highlighted in each panel.

A prime candidate involves internally generated decadal variability of SSTs in the tropical Pacific and Atlantic as noted above. Previous studies used climate model experiments with specified SSTs in those regions to document this teleconnection mechanism. We confirm those results here with a somewhat different climate model configuration where the model is in a “pacemaker” configuration^12^. In such a model set-up, tropical Pacific SSTs are specified to be those observed during the negative phase of the IPO and the rest of the model is fully coupled^13^ (Fig. S1a,b; see Methods). As seen in the earlier specified SST studies, the negative SST anomalies in the tropical Pacific produce an anomalous atmospheric teleconnection pattern with a ridge (positive 200 hPa height anomalies) over much of Europe, eastern Asia, and the North Pacific extending to the western U.S. (Fig. S1b) with significantly warmer surface temperatures over the western U.S. and Great Plains (Fig. S1a). There are also positive surface temperature anomalies over much of southern Europe and northern Asia. Comparing the surface temperature anomalies in Fig. S1a to the observations in Fig. 1b, warming is not nearly as widespread over North America in the model compared observations, and the significant large warming over Northern Europe is not present.

If the positive phase of AMV in the tropical Atlantic is specified in a pacemaker configuration (Fig. S1c,d) as it was for the Pacific (Fig. S1a,b), the Pacific responds with an opposite-sign SST anomaly response resembling the negative phase of the IPO^13^ (Fig. S1c). The combined effect of positive AMV and negative PDV produces a larger extratropical response that resembles some elements of an anomalous wave-5 pattern^14^. There are greatest positive 200 hPa height anomalies over southwestern North America and northern Asia (Fig. S1d), and large amplitude surface warming mainly over southwestern North America, southern and northwest Asia, Scandinavia, and parts of the Mediterranean region (Fig. S1c). This pattern also differs from the observations in Fig. 1b where largest amplitude warming covered nearly all of North America, northern Europe and parts of northeastern Asia. Thus, as seen in previous studies, parts of the warming pattern seen during the Dust Bowl years can be captured by either SST forcing from the tropical Atlantic, tropical Pacific, or a combination of both. It was noted above that the massive dust storms of the Dust Bowl intensified the drought over the Great Plains^9^ but that forcing likely had little effect on larger scale patterns involved with tropical SSTs in the Pacific and Atlantic.

Another forcing that we consider here for the first time, which could have further intensified the drought and heat of the Dust Bowl while communicating those effects to other areas of the Northern Hemisphere, involves the actual heating of the desiccated land surface of the Great Plains themselves. In a climate model sensitivity experiment where the land surface over the Great Plains is totally dried out (analogous to what happened during the Dust Bowl) and where sea surface temperatures (SSTs) are specified to climatology and thus cannot react to the forcing from the Great plains (see Methods), the effect of the hot and dry Great Plains can be isolated to quantify the contribution to the wave-5 teleconnection pattern that rings the Northern Hemisphere midlatitudes^15,16^ (Fig. 3b). This arises in the model experiment from the associated lower tropospheric heating over the Great Plains that, by itself through the circumglobal warm season atmospheric teleconnection pattern, produces anomalously warm conditions over parts of northern Europe, Russia, and northeastern Asia (Fig. 3a)^16^.

**Figure 3:**
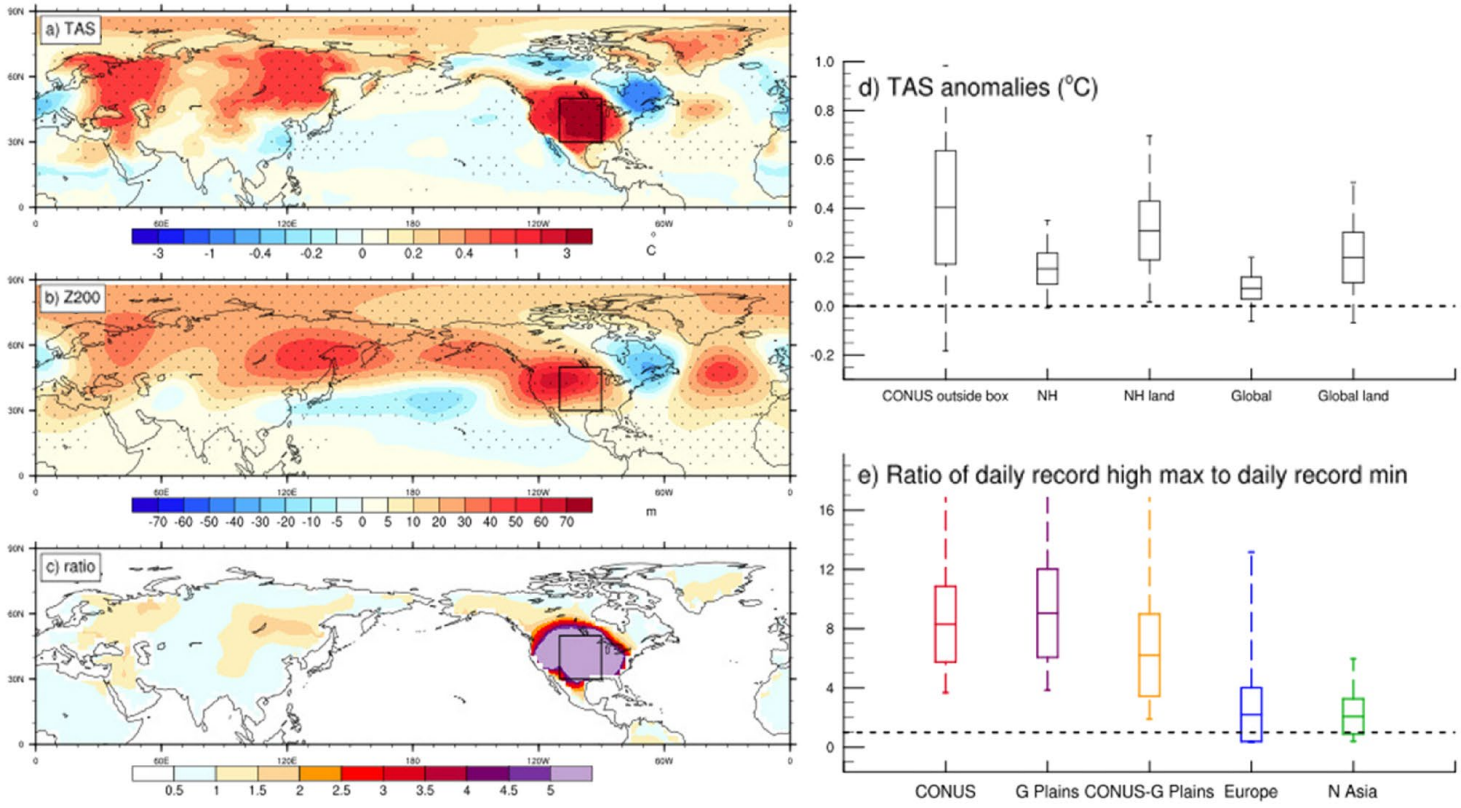
Dust Bowl heat extremes in the model experiment. The MJJA season average anomalies, experiment minus control, from the climate model sensitivity experiment where soil moisture over the Great Plains box (denoted in panels **a-c**) is set to zero; stippling indicates differences significant at the 95% level; (**a**) surface air temperature (TAS, °C); (**b**) 200 hPa height (Z200, m); (**c**) total records ratio differences; (**d**) box and whisker plots (markings indicate maximum value, + 1 standard deviation, mean, − 1 standard deviation, minimum value) for TAS anomalies (°C) for continental U.S. (CONUS) averaged outside the Great Plains box; NH (Northern Hemisphere), NH land (Northern Hemisphere land grid points), global average, global average over land grid points; (**e**) total records ratios averaged over various regions taken from the data shown in (**c**) for continental U.S. (CONUS), Great Plains, Great Plains minus CONUS, Europe (10° W–60° E, 30° N–50° N), and Northern Asia (80° E–160° E, 35° N–75° N) with box and whisker markings as in (**d**).

The anomalous heating over the Great Plains in the model experiment in the warm season produces surface temperature anomalies of + 4.4 °C over the Great Plains (Fig. 3a). But over the U.S. outside the Great Plains the mean temperatures are also significantly warmer with a positive temperature anomaly of + 0.40 °C (Fig. 3d). There is significantly greater surface warming over the Northern Hemisphere overall (+ 0.15 °C), and over Northern Hemisphere land as well (+ 0.31 °C) (Fig. 3d). Additionally, the global surface temperatures increase by + 0.07 °C while global land temperatures warm by + 0.20 °C, all from a regional drying and warming over the Great Plains in this model experiment. Such a warming from the Dust Bowl drought was one of the major contributors to early century large-scale warming^17^. The pattern of warming anomalies in the model experiment in Fig. 3a is not an exact match to the observed anomalies in Fig. 1b due to the contributions from the tropical Atlantic and Pacific to the teleconnection pattern as shown in Fig. S1 and discussed earlier. However, the similarities in the model to the observed pattern noted above indicate that the heating from the Great Plains made major contributions to the circumglobal teleconnection pattern and associated surface temperature anomalies over Northern Hemisphere land areas during the 1930s.

However, our focus here is heat extremes, and as could be expected from a previous study^11^, these increases in average surface temperature in areas around the Northern Hemisphere are reflected in larger records ratios and thus greater extreme heat (Fig. 3c,e). The biggest increases of the records ratio in the model experiment reach a value of about 9 over the Great Plains, but the value over the entire U.S. is about 8. There are increases in the ratio to values greater than one over most of North America, and specifically in the U.S. outside the Great Plains (Fig. 3c) with an average value of about 6 (Fig. 3e). In regions farther afield where mean warming from the Great Plains is transferred by the circumglobal wave 5 pattern in the model experiment (Fig. 3b), the records ratio values over Europe and northern Asia are both about 2. This indicates that there is an increase in heat extremes in those regions driven only by the dryness and heating over the Great Plains in the model (Fig. 3c,e).

The agents of these far-field increases of heat extremes driven from the Great Plains by the wave-5 teleconnection pattern can be represented by anomalies in vertical motion (Fig. 4a). From the intense surface heating over the Great Plains in the sensitivity experiment, there are mostly increases in upward vertical motion as could be expected (negative anomalies in Fig. 4a). But in surrounding regions of North America outside the Great Plains there is mostly anomalous downward vertical motion (positive anomalies). That intensified subsidence suppresses cloud formation resulting in decreases of cloud amount over the northwestern and northeastern U.S. and much of Canada (Fig. 4b). Additionally, the large anomalous ridge over Russia (positive 200 hPa height anomalies, Fig. 3b) is associated with anomalous downward motion there (Fig. 4a) with associated decreases in cloud (Fig. 4b). Those decreases in cloud allow more incoming solar radiation to reach the surface, and contribute to large increases of net surface heat flux in those regions (Fig. 4c). Positive sign anomalies in Fig. 4c indicate that more energy is reaching the surface to contribute to surface warming seen in those same regions (Fig. 3a). In this way dry surface conditions over the Great Plains region can heat the lower troposphere there, produce anomalies in vertical motion and clouds, along with warmer surface temperatures and increased heat extremes. These are all associated with the anomalous wave-5 circumglobal atmospheric circulation pattern driven by the Dust Bowl drought itself.

**Figure 4:**
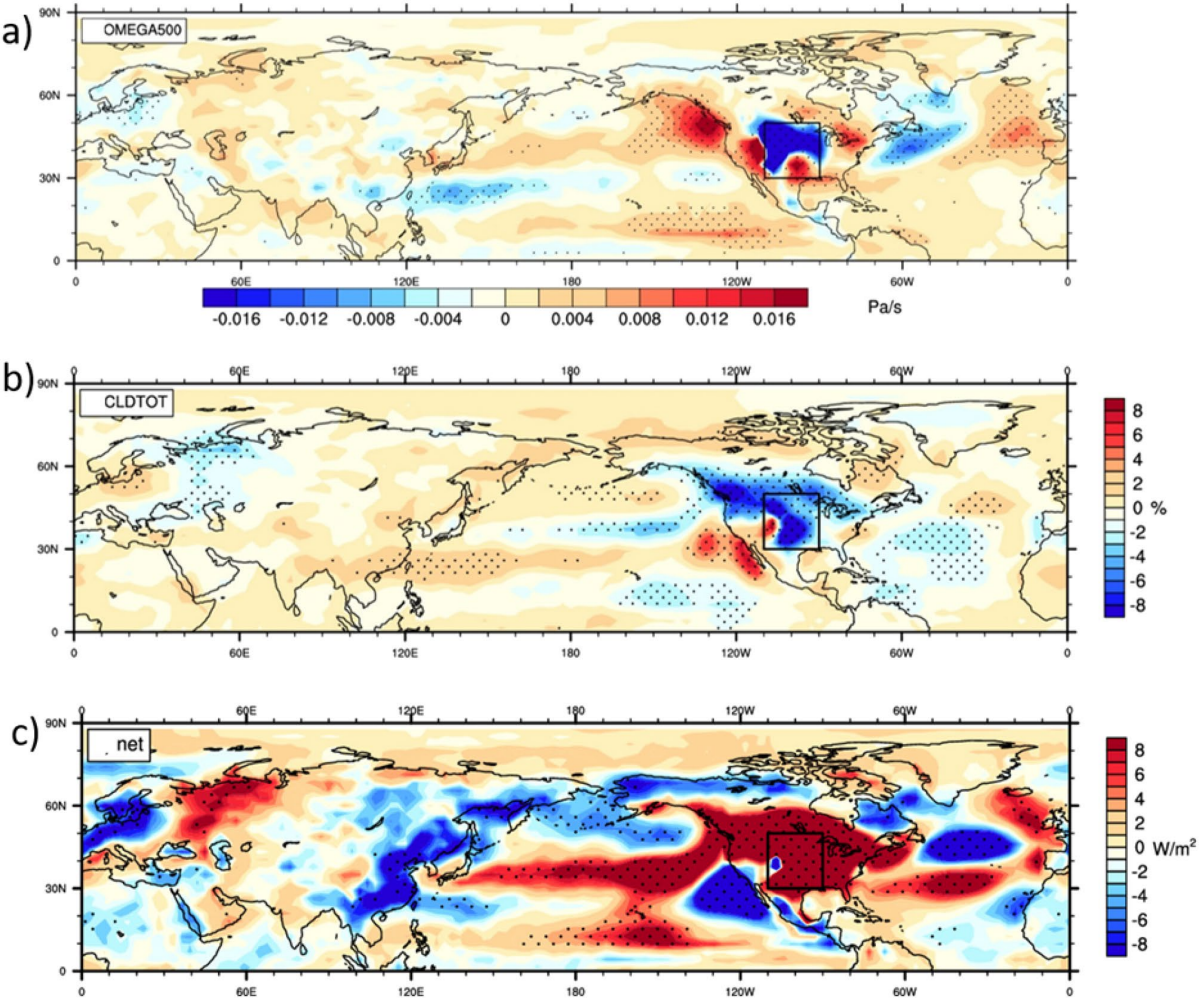
Processes producing far-field heat extremes. Anomalies from climate model experiment, experiment minus control, MJJA averages, stippling indicates differences significant at the 95% level; (**a**) vertical velocity at 500 hPa (Pa sec^−1^), positive values indicate anomalous downward vertical velocity; (**b**) total cloud (%), negative values indicate fewer clouds; and (**c**) net surface heat flux (W m^−2^), positive values indicate anomalous energy downward into the surface.

In other regions, the consequence of the anomalous wave-5 pattern is anomalous atmospheric advection anomalies that contribute to anomalously warm surface temperatures. Sea level pressure (SLP) anomalies in the model experiment show a heat low over the Great Plains, positive SLP anomalies across the North Pacific and North Atlantic, and negative anomalies over Northern Europe (Fig. S2a). The consequent lower level wind anomalies (Fig. S2b) show southerly component winds reaching north into Scandinavia and eastern Asia where there are also positive low level temperature advection anomalies (Fig. S2c). Thus, these low level atmospheric circulation anomalies, tied to the anomalous wave-5 teleconnection pattern, contribute to positive surface temperature anomalies over parts of Northern Europe, Scandinavia, and eastern Asia, with consequent increases of extreme heat (Fig. 3a,c).

## Discussion

Our results show that the extremely dry conditions over the Great Plains during the Dust Bowl drought of the 1930s, which was made worse by unprecedented and catastrophic land use practices, acted to intensify a naturally-occurring drought that was likely initiated by internally generated decadal timescale SST anomalies in the tropical Pacific and Atlantic. The resulting dust storms arising from the unusually dry surface also intensified the drought and heat over the Great Plains. A climate model experiment identifies a new source, lower tropospheric heating over the Great Plains from the anomalously dry conditions, that spreads the temperature extremes arising from the regional drought to other areas of the Northern Hemisphere. This newly identified forcing related to heat extremes produces an anomalous warm season wave-5 teleconnection pattern driven by the lower tropospheric heating over the Great Plains. The resulting anomalies in vertical motion, clouds, net surface heat flux, and temperature advection produce anomalous warmth in most of North America outside the Great Plains drought region, and in areas of northern Europe, Russia and parts of eastern Asia. That warmth is associated with anomalous heat extremes in the 1930s in those regions that stand out from the record and were not matched or exceeded until the twenty-first century when warming has mostly been forced by increasing GHGs.

A pacemaker model configuration run with the characteristics of anomalous SSTs that occurred on decadal timescales in the tropical Pacific and Atlantic in the 1930s confirms previous model results that the consequent teleconnection patterns spread anomalous warmth to some other parts of the Northern Hemisphere. But our model simulations show that the SST-forced circulation patterns cannot account for all of the areas of anomalous warmth of the 1930s. We show here for the first time that the teleconnections from the heating over the Great Plains themselves from the drought and land use conditions are necessary to account for the unprecedented heat extremes over large areas of the Northern Hemisphere in the 1930s that have only recently been approached or exceeded.

The model sensitivity experiment here is designed to provide a large forcing by totally drying out the Great Plains, and one could ask how realistic this forcing is compared to what actually occurred there during the Dust Bowl. While is it unlikely that the entire region had zero values of soil moisture, and it is impossible to know what the actual soil moisture values were during the Dust Bowl, anecdotal accounts from that time period point to vast expanses of bare denuded land where soil moisture likely approached extremely low values over huge areas of the Great Plains^1^. If surface temperature anomalies are an indication of relative dryness, warming of over 4 °C in the model experiment is about twice the observed warming of over 2 °C, suggesting the forcing in the model experiment could be about twice what was observed. But even with that level of warming, the circumglobal wave 5 pattern would emerge and provide the teleconnection mechanism to spread heat extremes to far-field areas of the Northern Hemisphere, and this connection between Great Plains soil moisture and the circumglobal wave 5 pattern is present in observations as well^16^. Thus this sensitivity experiment demonstrates not only a new mechanism that can spread heat extreme far beyond the source, but also that the model response is likely representing a key contributing factor to the conditions that existed over the Northern Hemisphere during that very anomalous period over the Great Plains. This points to the possibility that future more intense regional droughts could have a previously unrecognized hemispheric influence on heat extremes.

## Methods

### Climate model sensitivity experiment

The experiment^16^ involves setting the soil water over the Great Plains to zero in an atmosphere/land stand-alone configuration of the Community Earth System Model version 1 (CESM1)^18^ (often referred to as Community Atmospheric Model version 5,CAM5). It has 30 vertical levels and a horizontal resolution of roughly 1 degree. The land surface is fully coupled outside the Great Plains, and climatological SSTs are specified to long term monthly means from a fully-coupled long control run. There are 100 ensemble members run for the time period May through August, and these are compared to a 100 year control run with the same atmosphere/land initial conditions, taken from different years of the last 500 years of the 2600-year CAM5 control run^18^ and are at least five years apart. Averages are shown for the warm season when heat is most extreme, May–June–July–August (MJJA).

### Computing the records ratios

The records ratios from the model sensitivity experiment are computed by first accumulating the daily record high maximum temperatures and daily record low minimum temperatures at each location from the 100 year control run. The records ratio decays at the rate of 1/n where n is the year^10^. By the end of 100 years, the ratio is close to 1 as expected for a stationary climate. Then we compute the ratio for the 100 ensemble members of the sensitivity experiments compiled in relation to the last year of the control run. We then produce geographic plots of that ratio for the Northern Hemisphere and averages of the records ratios over various regions (Fig. 3). For the observations, we sum the number of daily record high maximum temperatures (at each station in the observations) for each year averaged by country, and also sum the number of daily record low minimum temperatures in the same way. We then compute the ratio of the sum of the record highs divided by the sum of record lows, and form the ratio averaged by country and by year. We then perform decadal averages of the ratio.

### Pacemaker model experiments

These are described by Meehl et al. (2020)^13^ where the internal component of the observed Pacific and Atlantic decadal-timescale variability is separated from the externally-forced part^19^. The internally generated SST components are obtained as the residuals of the observed North Atlantic and Pacific average SSTs (resembling the AMV/AMO and PDV/IPO) for 40° S to 60° N and Equator to 60° N, 75° W to 7.5° W, respectively, after subtracting the externally-forced component. The AMV and PDV spatial patterns of SST are then calculated by regressing the annual-mean observed SST time series onto the respective AMV and PDV indices. These index time series and the SST fields are low-pass filtered for the period 1870–2013 prior to the regressions using a Lanczos filter with a 10-year cutoff period using 21 weights. The global coupled climate model version used in the pacemaker experiments is the CESM1-CAM5 model (hereafter CESM1) that is the same model described in the CESM Large Ensemble Project^18^; the atmospheric component is used in the sensitivity study described above. All components have approximately 1° horizontal resolution. The ocean has 60 levels in the vertical and a meridional mesh refinement down to a quarter of degree near the equator, while the atmospheric component has 30 hybrid vertical levels.

Two sets of 30-member ensemble simulations are run for the idealized specified PDV and AMV configurations. All external forcings are held constant at pre-industrial values. One set consists of simulations for AMV + and AMV − , the other set has the simulations for PDV + and PDV − . Each experiment is run for 10 years during which the respective specified SST anomalies are kept constant in time while the rest of the model is fully coupled. The 30 ensemble members for each experiment are formed by introducing round-off perturbations in the initial atmospheric temperature.

## Supplementary Information

Supplementary Information.

## Data Availability

GISTEMP surface temperature data^20^ (Lenssen et al. 2019) are available from https://data.giss.nasa.gov/gistemp/. BEST surface temperature data^21^ (Cowton et al. 2019) are available from https://climatedataguide.ucar.edu/climate-data/global-surface-temperatures-best-berkeley-earth-surface-temperatures. Observed daily record high maximum temperatures and record daily record low minimum temperatures are from NCEI and available at https://www.ncdc.noaa.gov/cdo-web/datatools/records.

## Code availability

Previous and current CESM versions are freely available at www.cesm.ucar.edu:/models/cesm2/. The CESM solutions / datasets used in this study are also freely available from the NCAR Digital Asset Services Hub (DASH) at data.ucar.edu or from the links provided from the CESM web site at www.cesm.ucar.edu.
