# Supplementary material for: How the Great Plains Dust Bowl drought spread heat extremes around the Northern Hemisphere
Source: Sci Rep. 2022 Oct 17;12:17380. doi: 10.1038/s41598-022-22262-5 (PMC9576710; doi:10.1038/s41598-022-22262-5)
Supplement: Supplementary file 1 — Supplementary Information. [file 41598_2022_22262_MOESM1_ESM.docx]

Supplementary Material

**How the Great Plains Dust Bowl Drought spread heat extremes around the Northern Hemisphere**

Gerald A. Meehl^1*^, Haiyan Teng^2^, Nan Rosenbloom^1^, Aixue Hu^1^,

Claudia Tebaldi^3^, and Guy Walton^4^


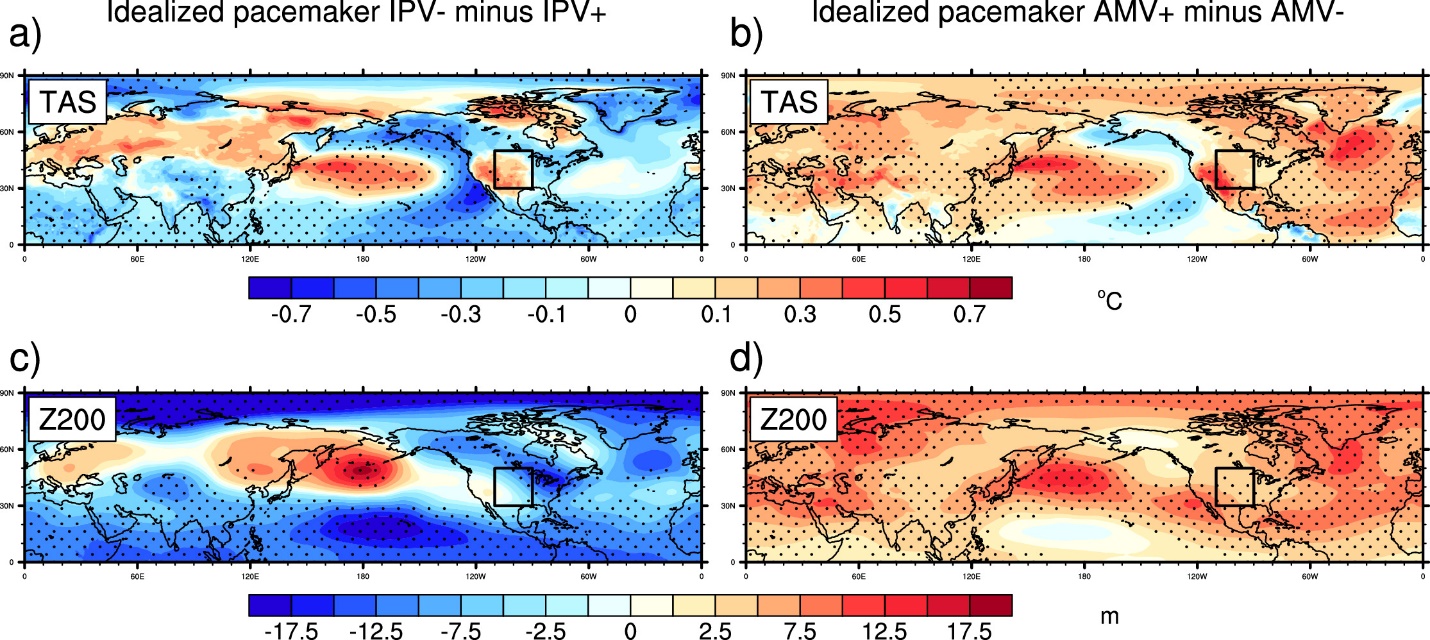


**Figure S1**: Results from idealized pacemaker experiments (see Methods), where panels a and b are for the negative interdecadal Pacific variability (IPV-) minus positive IPV (IPV+) experiments, and c and d are from the positive Atlantic Multidecadal Variability (AMV+) minus negative AMV (AMV-), stippling indicates differences significant at the 95% level, for a) surface air temperature (TAS, °C); b) 200 hPa height (m); c) same as (a); and d) same as b.


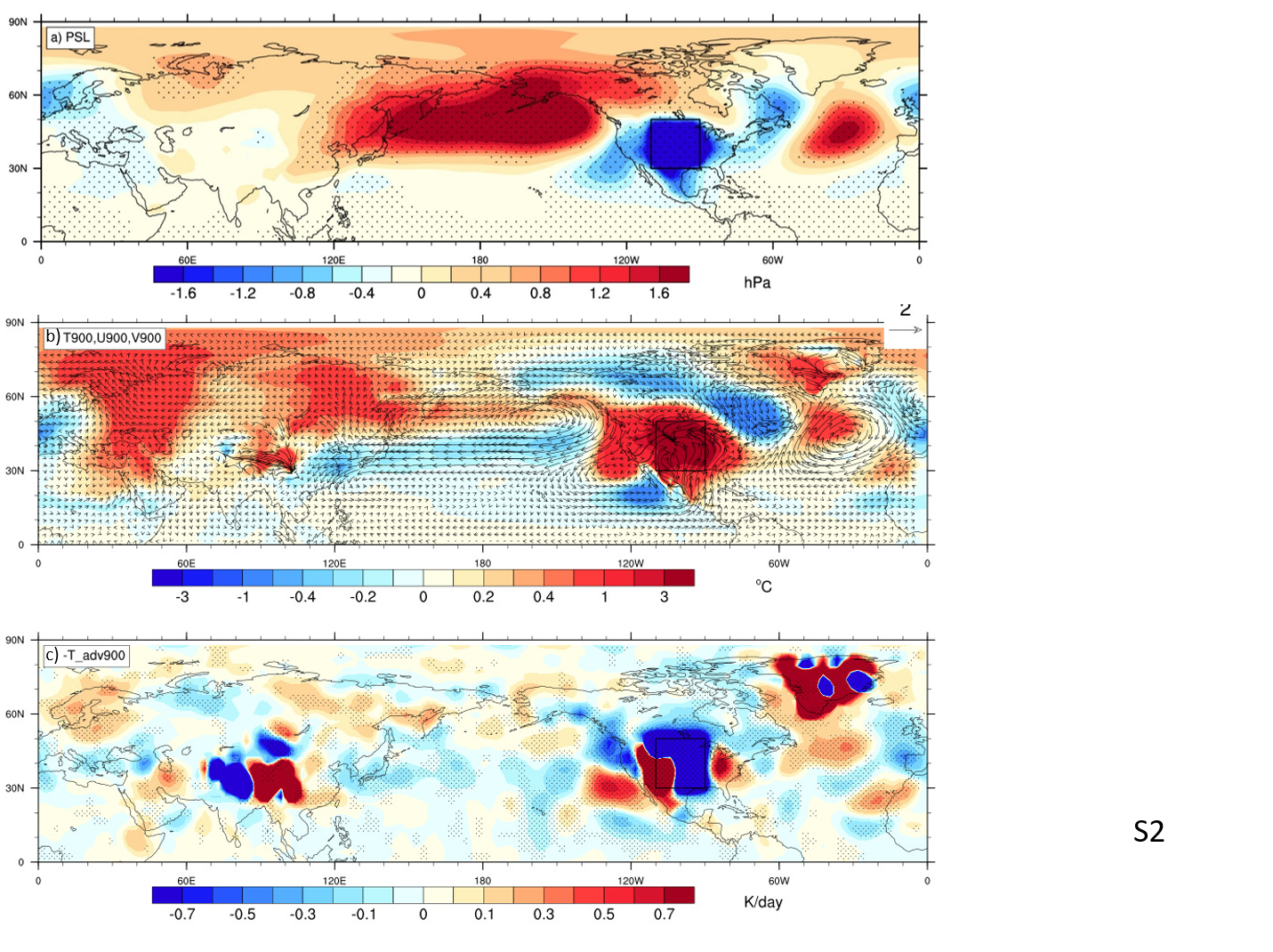


**Figure S2**: Same as Fig. 4 except for a) sea level pressure (PSL, hPa), b) temperature at 900 hPa (colors, °C), and vector winds calculated from u and v component winds at 900 hPa (scaling vector at upper right, 2 m sec^-1^); c) 900 hPa temperature advection (K day^-1^).
